# Low sensitivity of the COVID-19 antigen test (PANBIO™ COVID-19 Ag rapid test) to detect asymptomatic infections in health personnel of the National Institute of Respiratory Diseases

**DOI:** 10.3389/fmed.2022.977924

**Published:** 2022-09-14

**Authors:** Eduardo Becerril Vargas, Gabriel Cojuc-Konigsberg, Mario Alberto Mujica Sánchez, María Del Carmen García Colín, Daniel Alfredo Camacho Corral, Hugo Hansel Chávez Morales, José Nicolas Aguirre Pineda, Eduardo Martínez Bravo, Alejandro Ortiz Martínez, José Arturo Martínez Orozco, Victor Manuel Rodríguez-Sánchez, Jesús Ariel Mariscal Ochoa, Brian Pantoja Jiménez, Israel A. Morales Lozada, Andrea Iraís Cuevas Rodriguez

**Affiliations:** Clinical Microbiology Laboratory, National Institute of Respiratory Diseases, Mexico City, Mexico

**Keywords:** COVID-19, rapid antigen tests, asymptomatic, detection, PCR

## Abstract

**Background:**

COVID-19 requires an early diagnosis to optimize management and limit transmission. SARS-CoV-2 is able to spread effectively. Infected asymptomatic individuals have been found to be contagious. RT-qPCR is the currently recommended laboratory method for diagnosing acute infection. However, rapid antigen detection (RAD) tests are not only fast, but require less specialized training. The possibility of using RAD tests to identify asymptomatic patients is attractive, as it could effectively contribute to minimizing the hospital spread of SARS-CoV-2. The objective of the study was to determine the performance of RAD vs. RT-qPCR for the detection of asymptomatic cases in INER health personnel.

**Methods:**

In order to follow WHO guidelines, generalized tests, a test station for health care workers was implemented on demand. A rapid test was carried out and a second sample was taken to be processed by RT-qPCR. With the results of both tests we conducted a retrospective study. Sensitivity, specificity, positive predictive value, negative predictive value and negative likelihood ratios were calculated.

**Results:**

A total of 1640 RAD tests were performed in health care workers (mean age was 39, 69, 47% with a self-reported comorbidity). Participants provided 1,640 valid RAD/RT-qPCR test pairs with 2% testing positive *via* RT-qPCR. 12 RAD samples were positive for SARS-CoV-2. Overall sensitivity of the PANBIO ™ COVID-19 Ag Rapid Test test was 35.2%.

**Conclusions:**

RADs are not recommended for the detection of asymptomatic cases due to low performance.

## Introduction

The pandemic caused by SARS-CoV-2 remains a global challenge. Although the initial identification and subsequent implementation of diagnostic methods was extremely rapid, the pandemic could not be contained, unlike the 2002–2003 SARS-CoV-1 outbreak. One reason for this is the efficient spread of SARS-CoV-2, in part because of asymptomatic individuals who are able to transmit the virus without being detected. Due to the magnitude of the pandemic, the World Health Organization (WHO) has recommended widespread testing for SARS-CoV-2 (1–3).

Early diagnosis plays a crucial role in reducing the transmission chain of SARS-CoV-2 and in the timely clinical management of COVID-19. RT-qPCR is currently the recommended laboratory method to diagnose acute SARS-CoV-2 infection. Several factors limit the use of these time-consuming technique, such as preparation of working solutions, extraction and transfer of nucleic acids to the amplification device, and having trained personnel, (4). Due to the variable performance observed between different RT-qPCR assays, it is currently recommended to repeat the test in patients with intermediate or high clinical suspicion of COVID-19 infection when the initial result is negative (5–7).

The recommendation of extensive and repeated testing, as well as rapid viral spread throughout the world are some components that have exponentially increased the number of performed RT-qPCR. High test volumes pose important challenges for clinical laboratories, particularly regarding equipment and personnel (8).

Multiple tools have been developed for the rapid detection of SARS-CoV-2 in order to streamline testing. Rapid antigen detection (RAD) tests that qualitatively detect SARS-CoV-2 antigens are currently available. RAD tests detect viral antigens using SARS-CoV-2 antibodies that are coated and immobilized on a device. RAD test results can be interpreted without a specialized instrument and are available in < 30 min. Therefore, RAD tests can decrease the workload in hospitals and diagnostic laboratories and improve turnaround time and reagent restrictions for PCR processing. Nonetheless, RAD tests are currently recommended for confirmation of symptomatic cases, with limited evidence for detection in asymptomatic people. A sensitivity of 50–90% has been reported, so it is important to continue evaluating the performance of these diagnostic tests (9).

The objective of the study is to evaluate the diagnostic utility of the Abbott PANBIO™ COVID-19 Ag test in asymptomatic individuals in a high-exposure environment. The aforementioned test is a lateral flow colorimetric immunochromatographic assay, which contains specific antibodies for the SARS-CoV-2 antigen. When the antigen is present, a visible black band appears on the test line as a result of antigen-antibody complex formation. The result is obtained in 5 to 8 min.

## Materials and methods

A retrospective observational study was performed. All asymptomatic National Institute of Respiratory Diseases (INER) health workers who attended the testing module and underwent both a rapid Ag ABBOTT PANBIO^TM^ test and an RT-qPCR from December 2020 to May 2021 were included.

Patients who developed symptoms after obtaining a positive RT-qPCR and/or Rapid Test and patients without a case notification in the National Epidemiological Surveillance System (SISVER-SIVANE) were excluded.

The results of rapid antigen detection tests (RAD) and RT-qPCR performed on health personnel were gathered and reviewed. With the obtained information a database was created to conduct a statistical analysis. The clinical data of the patients was obtained from the Epidemiological Surveillance System for Respiratory Diseases.

Statistical analysis was performed using the statistical package SPSS 27. Results are presented through descriptive statistics. Medians with ranges and/or means with standard deviations were used for quantitative variables; for qualitative data, frequencies and percentages were used.

The following were calculated:

- Sensitivity: defined as the number of true positive results divided by the sum of the true positive and false negative results.
- Specificity: defined as the number of true negative results divided by the sum of true negative and false positive results.
- Positive predictive value (PPV): defined as the number of true positives divided by the sum of the true positive and false positive results.
- Negative predictive value (NPV): defined as the number of true negatives divided by the sum of the true negative and false negative results.
- Negative likelihood ratio (LR-): defined as sensitivity divided by 1 minus specificity.

### Ethical considerations

This study follows the ethical guidelines established for the use of patient information and has been approved by INER's ethical committee.

## Results

**Figure 1:**
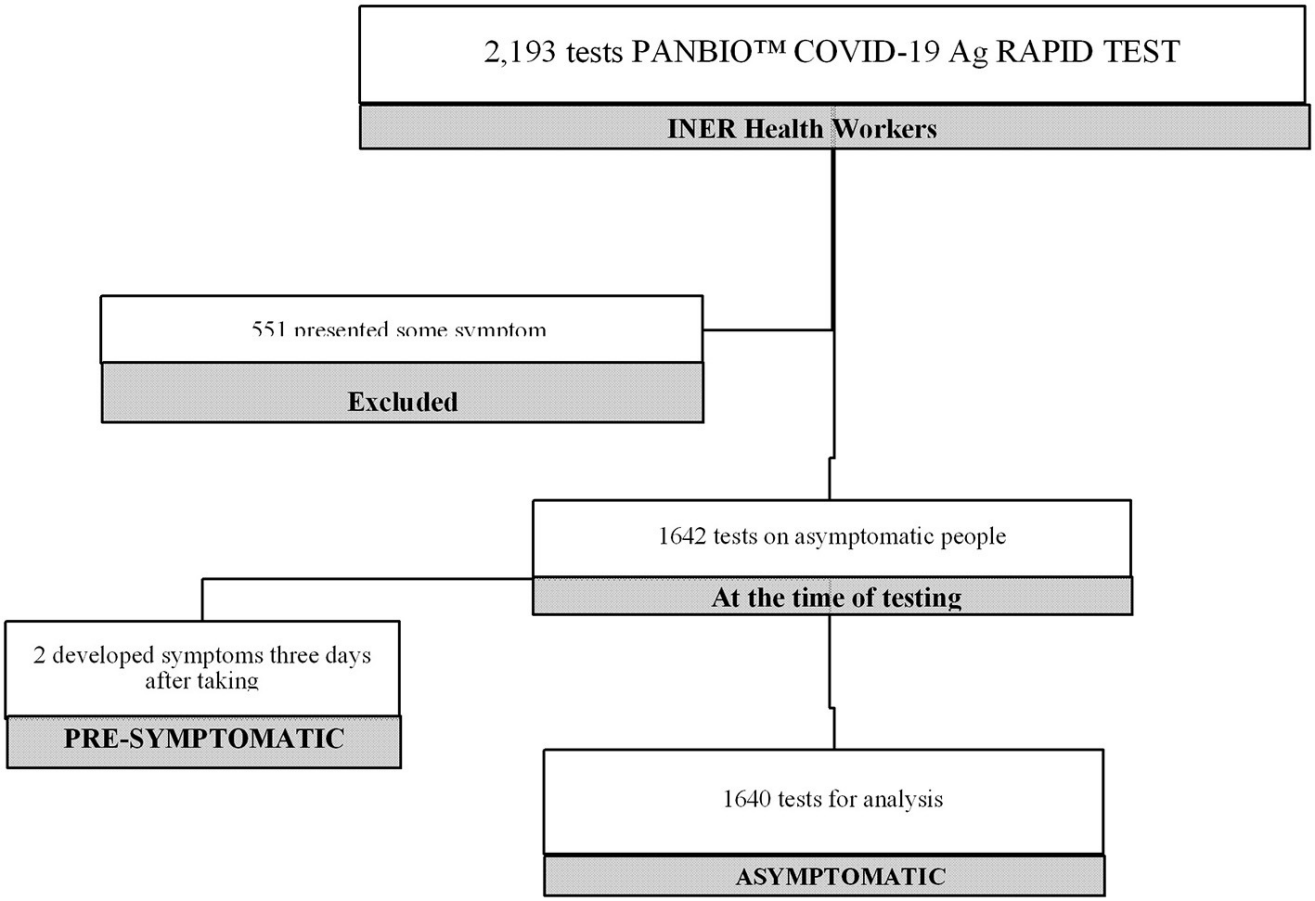
Number of tests performed in the study.

A total of 2,193 rapid tests were performed on health personnel during the study period. 551 tests were excluded since the subjects presented symptoms at the time of taking the nasopharyngeal sample; 1,640 test results were included in the study. The tests were performed on 1080 INER workers (Figure 1).

**Figure 2:**
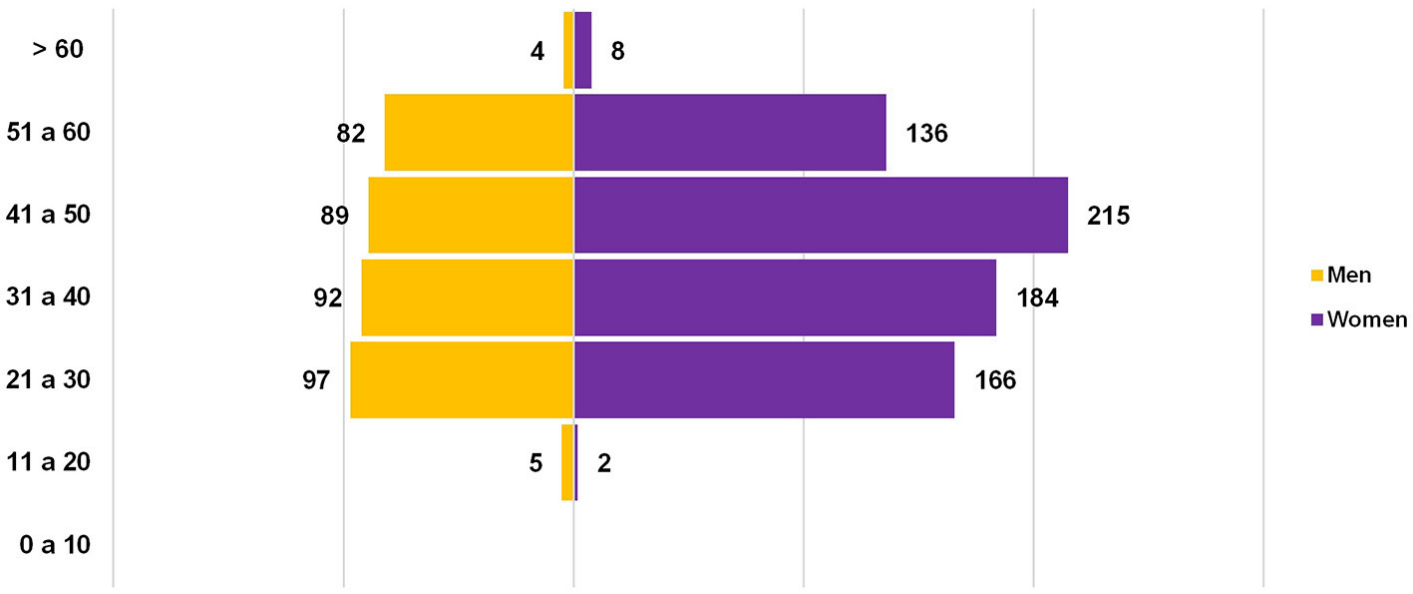
Distribution of INER staff by age and gender.

Mean age was 39.69 ± 11.03 years, 64% (1052/1080) were women (Figure 2). 41% (442/1080) of the evaluated health personnel were nursing personnel and people with administrative activities and 14% (151/1080) were medical staff (Table 1).

**Table 1:** Designations of the tested personnel.

**Designation**	***N***	**%**
Nursing staff	224	21%
Admistrative staff	218	20%
Investigation staff	155	14%
Medical staff	151	14%
Chemicals	128	12%
Housekeeping/maintenance staff	73	7%
Technicians	55	5%
Feeding	36	3%
Social work	25	2%
Stretchers	15	1%

47% (508/1080) of the health personnel included in our study had at least one comorbidity. Obesity, overweight and systemic arterial hypertension (SAH) were the most frequent associated comorbidities in included subjects (Table 2).

**Table 2:** Frequency of comorbidities in the patients included for the analysis.

**Comorbidities**	***N* (1080)**	**%**
Overweight	227	21%
Obesity	139	13%
Hypertension	62	6%
Diabetes	43	4%
Asthma	39	3,6%
Allergic rhinitis	23	2%
Hypothyroidism	22	2%
Cns diseases	10	1%
Others	22	2%

Of the 1,640 samples, 34 (2%) were reported positive by RT-1PCR and the rest were negative for SARS-CoV-2. 12 (0.7%) RAD tests were positive for SARS-CoV-2. Health personnel not related to direct patient care, such as administrative personnel and chemists, had the highest rates of positive tests. The highest number of asymptomatic carriers were reported in chemists, nurses and doctors (Table 3).

**Table 3:** Frequency of asymptomatic infections.

**Designation**	**SARS-CoV-2**	**%**	**Total**
Chemicals	8	8%	98
Admistrative staff	3	6%	51
Medical staff	5	2%	223
Nursing staff	7	2%	338
Stretchers	5	2%	262
Housekeeping/maintenance staff	4	1%	303
Research staff	2	1%	232

For the detection of SARS-CoV-2, sensitivity through the rapid antigen detection test was 35.2% and specificity was 100%, with a PPV of 100%, an NPV of 98.6% and LR- of 0.65 (Table 4).

**Table 4:** Comparison between RT-PCR performance (reference method) and PANBIO COVID-19 Ag Rapid test.

**PANBIO™ COVID-19 Ag RAPID TEST**	**RT-PCR (reference method)**		**Sensitivity** **(%)**	**Specificity (%)**	**PPV (%)**	**NPV (%)**	**LR (-)**
	**Positive**	**Negative**					
Positive	12	0	35.2	100	100	98.6	0.65
Negative	22	1606					

NPV, negative predictive value; PPV, positive predictive value; LR(-), Likelihood Ratio for a negative test.

## Discussion

This study was designed to compare the performance of the PANBIO™ COVID-19 Ag test with RT-QPCR -the current gold standard. for the identification of health workers infected with SARS-CoV-2 who were asymptomatic.

Sensitivity through rapid antigen detection test was 35.2%. The performance obtained in the study for the detection of asymptomatic carriers was lower when compared with the existing evidence on asymptomatic patients reported by Fenollar et al. (45.4%) (10), Torres et al. (48.1%) (11), Linares et al. (54.5%) (12) and Bulilette et al. (59%) (13). However, in contrast with our study, these protocols were carried out in people who were in contact with a positive symptomatic patient with a testing timeframe of < 7 days after exposure, and were not carried out in an environment with constant exposure to the virus such as healthcare workers.

Improvement in antigen test performance is required because of the potentially large number of false negatives due to low sensitivity, despite the high specificity of the assay. Nevertheless, the PANBIO ™ COVID-19 Ag Rapid test has several benefits over RT-qPCR for SARS-CoV-2 detection, such as simplicity of use, easy availability, low cost, and a short time needed to obtain the results.

The prevalence of asymptomatic patients in our study was 2%, which to our knowledge is much lower than that reported in different meta-analyses conducted to date, ranging from 15.6 to 35.1%14, (15). The low number of asymptomatic carriers at INER can be explained by the intense training given to all staff for the proper use of personal protective equipment (16).

The viral load of SARS-CoV-2 reaches its peak around the time of symptom onset (14). This information it is still uncertain for asymptomatic individuals. Peak viral loads are particularly relevant when studying RNA viruses, since an exponential phase of replication has been described, which can go from undetectable levels to millions in a day, and could be useful as a threshold in asymptomatic subjects (17). Further studies could be conducted in order to determine the ideal time for testing.

Among the strengths of the current study is that it reflects the performance of the rapid antigen test in a real-life environment, where high exposure to COVID-19 is encountered. Among its limitations is the lack of knowledge of specific time between exposure and testing. Therefore, it could be determined if the test was performed too soon or too late.

## Conclusions

The use of rapid antigen tests is not recommended to identify people with SARS-CoV-2 infection who are asymptomatic and should not be used in ruling out diagnoses in these individuals. In populations with high disease prevalence, rapid antigen tests could be utilized for screening programs and ruling in confirmed cases.

## Data availability statement

The raw data supporting the conclusions of this article will be made available by the authors, without undue reservation.

## Ethics statement

The studies involving human participants were reviewed and approved by Comité de Ética en Investigación del Instituto Nacional de Enfermedades Respiratorias Ismael Cosío Villegas. Written informed consent for participation was not required for this study in accordance with the national legislation and the institutional requirements.

## Author contributions

Conception and design of study: EB, GC-K, and JA. Drafting of manuscript and critical revision: EB, GC-K, JA, EM, AO, and MA. Data analysis and interpretation: EB, EM, and AO. Acquisition of data, laboratory, and clinical: DA, JA, HH, BP, VR-S, and MA. Approval of final version of manuscript: MA, EB, and GC-K. All authors contributed to the article and approved the submitted version.

## Conflict of interest

The authors declare that the research was conducted in the absence of any commercial or financial relationships that could be construed as a potential conflict of interest.

## Publisher's note

All claims expressed in this article are solely those of the authors and do not necessarily represent those of their affiliated organizations, or those of the publisher, the editors and the reviewers. Any product that may be evaluated in this article, or claim that may be made by its manufacturer, is not guaranteed or endorsed by the publisher.
